# Hospital variation in surgical outcomes for gastric cancer: the impact of case-mix and treatment across a global cohort

**DOI:** 10.1007/s10120-025-01696-6

**Published:** 2026-01-13

**Authors:** Sander J. M. van Hootegem, Margrietha van der Linde, Marcel A. Schneider, Jeesun Kim, Felix Berlth, Yutaka Sugita, Peter P. Grimminger, Gian Luca Baiocchi, Giovanni De Manzoni, Maria Bencivenga, Suzanne S. Gisbertz, Souya Nunobe, Han-Kwang Yang, Christian A. Gutschow, Sjoerd M. Lagarde, Hester F. Lingsma, Bas P. L. Wijnhoven, The GastroBenchmark & GASTRODATA consortiums

**Affiliations:** 1 Department of Surgery, Erasmus Medical Center, University Medical Center, P.O. Box 2040, 3000CA Rotterdam, The Netherlands; 2 Department of Public Health, Erasmus Medical Center, Rotterdam, The Netherlands; 3 Department of Surgery and Transplantation, University Hospital Zürich, Zürich, Switzerland; 4 Department of Surgery, Seoul National University Cancer Hospital, Seoul, South Korea; 5 Department of General-, Visceral- and Transplant Surgery, University Medical Center Mainz, Mainz, Germany; 6 Department of Surgery, University Hospital of Tübingen, Tübingen, Germany; 7 Department of Gastroenterological Surgery, Cancer Institute Hospital of the Japanese Foundation for Cancer Research, Tokyo, Japan; 8 Department of Clinical and Experimental Sciences, University of Brescia, Brescia, Italy; 9 Department of Surgery, University Hospital of Verona, Verona, Italy; 10 Department of Surgery, Amsterdam UMC location University of Amsterdam, Amsterdam, The Netherlands; 11 Cancer Treatment and Quality of Life, Cancer Centre Amsterdam, Amsterdam, The Netherlands

**Keywords:** Stomach neoplasms, Gastrectomy, Treatment outcome, Quality of health care

## Abstract

**Background:**

There is substantial global variation in demographics, disease burden, and treatment for gastric cancer patients. Benchmarking is an instrument to assess such variation and enables to investigate to which extent case-mix and treatments explain differences in outcomes. We aimed to evaluate hospital-level variation in surgical outcomes following gastrectomy for gastric cancer before and after adjusting for case-mix and treatment-related factors.

**Methods:**

Data were retrieved from the GastroBenchmark and GASTRODATA databases, including consecutive gastric cancer resections performed between 2017 and 2021 from 43 centers. Patients who underwent a (sub)total gastrectomy for adenocarcinoma were identified. Outcomes included 30-day mortality, severe complications (Clavien-Dindo grade ≥ 3a), > 15 lymph nodes retrieved, negative resection margin (R0), prolonged hospitalization (> 14 days), readmissions (< 30 days), reoperations, and escalation of care. We assessed absolute inter-hospital variation for outcomes, and estimated outcomes using mixed-effect logistic regression models with a random intercept. We estimated crude, case-mix adjusted, and case-mix and treatment adjusted hospital effects. The conditional and marginal pseudo-R^2^ were used to quantify the variance in outcome explained by case-mix and treatment-related factors.

**Results:**

A total of 7818 patients from 41 hospitals were included, with contributions ranging from 12 to 2554 patients per hospital (IQR: 49–146). Observed 30-day mortality and severe complications ranged from 0 to 9.7% (IQR: 3.2%) and 5.3 to 31% (IQR: 7.7%), respectively. Larger variation between hospitals was observed for retrieval of > 15 lymph nodes (IQR: 12.3%), prolonged hospitalization (IQR: 14.4%) and readmissions (IQR 11.3%). This variation was reduced in the crude model, while adjusting for case-mix and treatment-related factors did not significantly reduce variation for any outcome. Case-mix factors had a limited contribution to the explained variance, except for 30-day mortality (33.9%) and negative resection margins (31.7%). Adding treatment-related factors increased the explained variance for 30-day mortality by 40.8%, but had low impact (< 10%) on the variance in most surgical outcomes.

**Conclusions:**

Case-mix and treatment factors are not the primary drivers of variation in surgical outcomes following gastrectomy. Case-mix adjustment can improve the validity of global comparisons for 30-day mortality, but does not seem essential for comparing other investigated outcomes.

**Supplementary Information:**

The online version contains supplementary material available at 10.1007/s10120-025-01696-6.

## Introduction

Gastric cancer (GC) ranks as the 5th most prevalent cancer and the 3rd leading cause of cancer-related mortality worldwide [1]. Surgery, with or without perioperative chemotherapy, remains the cornerstone of curative treatment. However, the complexity of a gastrectomy requires training and specialized skills, reflected by a learning curve and the association between case volume and surgical outcomes [2, 3]. Internationally, there is an increasing demand for monitoring and benchmarking the quality of surgical care at the hospital level. Various stakeholders, including government agencies, insurance companies, medical specialist associations, and patient organisations seek transparency on quality of care, in particular for low-volume and complex procedures [4–7]. In response, several projects have been initiated over the years to provide benchmark data for surgical cancer care [4, 6, 7]. Within this context, the GastroBenchmark and GASTRODATA consortiums were established, providing reference data for oncological gastrectomies derived from 43 centers in sixteen countries [8].

To serve its purpose, benchmark data should satisfy several conditions. One of these is that they should contain a large case-mix (i.e. patient and disease characteristics), reflecting a range of case complexity to represent real-life practice and enable unbiased evaluation [9]. For gastric cancer, it is well known that there are marked differences in demographics, disease burden and treatments between health care providers and regions [10, 11]. These differences may explain variation in surgical outcomes across hospitals, rather than solely being determined by the quality of surgical and perioperative care. Neglecting case-mix and treatment differences could lead to misleading or uninformative comparisons, defeating the purpose of benchmarking. However, the impact of case-mix and treatment approaches when comparing gastrectomy outcomes on a global scale remains unclear. Therefore, to assess the impact of these characteristics, we aimed to study the variation of surgical outcomes, both before and after adjusting for case-mix and treatment-related factors in a global cohort.

## Methods

### Patients and ethics

Patients were identified from the dataset collected as part of the GastroBenchmark and GASTRODATA collaborative, which includes data of 43 centers from sixteen countries, comprising consecutive patients who underwent an oncological gastric resection between 1st January 2017 and 31st December 2021 (Supplement 1). Further details on data collection have been previously described [8]. All patients with gastric adenocarcinoma who underwent an elective subtotal or total gastrectomy were eligible for inclusion. Patients in whom no Roux-en-Y or Billroth reconstruction was created, or no lymphadenectomy was performed were excluded.

The study committees of both consortiums approved the present study and provided the data for this analysis. Before the start of this study ethical approval was obtained in the Erasmus MC (MEC-2023-0696) and upon initiation of the database, approval was obtained in all participating centers.

### Outcomes, case-mix and treatment characteristics

Outcomes assessed were > 15 lymph nodes retrieved; negative resection margin (R0); severe complications (Clavien-Dindo ≥ 3a); re-operations; escalation of care; >14 days of hospital admission; readmission within 30 days and 30-day mortality. Escalation of care was defined as unplanned readmission to a higher surveillance unit.

Based on expert opinion, literature and data availability, the following factors were included as case-mix factors: age, gender, body mass index, American Society of Anesthesiologists (ASA) score, number of comorbidities (none, one, multiple), previous abdominal surgery, tumor location and T- and N-category. Treatment-related factors included administration of neoadjuvant treatment, extent of lymphadenectomy (D1(+), D2(+), D3), total or subtotal gastrectomy, type of access (open, minimally invasive and robot-assisted), and conversion.

### Statistical analysis

Missing data of baseline characteristics were imputed using multiple imputation then deletion with chained equations (five iterations) [12]. This means that all outcomes were included in the imputation regression model and imputed where missing, and subsequently, patients with initially missing outcomes were excluded in further analyses for that particular outcome.

We used mixed-effect multivariable logistic models to estimate the probability of a given outcome for each individual patient. Due to differences in case load and guidelines which the hospitals are subject to, a random intercept for hospitals was used to account for variation not captured in the covariates (i.e. unmeasured inter-hospital variation and random chance). Hospitals logging ten or fewer patients were excluded to improve representativeness of the data and limit the chance of bias, while ensuring statistical stability of the models Nonlinearity of continuous variables was addressed with restricted cubic splines (3 knots). Three models were used per outcome to assess variation: a crude model (random intercept for hospital only), a case-mix adjusted model, and a final model adjusted for case-mix and treatment-related factors. Models were tested with specific comorbidities (e.g. cardiovascular disease, pulmonary disease, endocrinological including diabetes, renal insufficiency and immunological disorders), but as this did not improve the Akaike Information Criterion (AIC) compared to simpler models using the number of comorbidities, we opted for the latter to preserve degrees of freedom without compromising model performance.

Observed variation between hospitals was displayed by calculating medians, range and interquartile range (IQR) on a hospital level. Crude and adjusted estimated probabilities with 95% confidence intervals of outcomes per hospital were presented in forest plots. The conditional pseudo-R^2^ was used to quantify the explained variance in outcomes by the models [13]. This measure reflects the explained variance in outcomes of the models by the fixed effects (i.e. the included variables) and random intercept, with 1 being the highest achievable score, representing 100% explanation of the variance. The marginal pseudo-R^2^ was calculated, reflecting the variance explained by the fixed effects alone, to assess the proportion of variance explained by the included variables in each model [13]. 

Meaningful inter-hospital comparison of outcomes rely on sufficient overlap in case-mix characteristics, such that patient populations treated in different hospitals have a shared range of of these variables [14]. For instance, if a specific category of a case-mix variable is absent in one center, the corresponding regression coefficient cannot be estimated. We therefore evaluated the case-mix balances across centers by estimating the probability of treatment in each center using logistic regression models, with hospital as the outcome and all case-mix variables as predictors. This led to sensitivity analyses in which we excluded patients from the two large Asian centers given their large case load (> 1000 patients per center) and distinct case-mix characteristics. Statistical analysis was performed in R version 4.3.2 (R Core Team, R Foundation for Statistical Computing, Boston, MA, USA) using the ‘lme4’, ‘rms’, ‘mice’, ‘mitools’ and ‘MuMin’ packages.

## Results

A number of 9662 patients were identified in the database, of which 7829 patients were eligible for inclusion. Two of the 43 hospitals had a case load of less than ten patients, resulting in a total of 7818 patients from 41 hospitals included in the analysis (Supplementary Fig. 1). The total case load per center ranged from 12 to 2554 patients, with a median of 81 patients per hospital (IQR: 49–146).

There was large variability in case-mix characteristics and treatment across hospitals (Table 1). The mean age of patients was 65 years, with a range from 55 to 76.5 years (IQR: 5) per hospital and 26.5–57.9% (IQR: 9.6%) being female. The percentage of patients with an ASA 1–2 score varied per hospital from 3.5 to 100% (IQR: 26.8%), while the percentage of patients with multiple comorbidities ranged from 10.3 to 28.9% (IQR: 18.6%). The proportion of patients with cT3-4 tumors ranged from 53.1 to 70.3% (IQR: 17.2%), and those with clinical positive nodal disease (cN1-3) from 36.8 to 58.9% (IQR: 22.1%).

**Table 1:** Case-mix and treatment characteristics

Variable	Total	Per hospital				Missing
	*n* (%)	Median (range)	Q1	Q3	IQR	
Patients/resections, *n*	7818 (100%)	81 (12–2554)	49	146	97	0
Age, years [mean (SD)]	65.0 (12.4)	67 (55–76.5)	65	70	5	9
Female (vs. male)	2842 (36.4%)	37.8% (26.5–57.9)	32.4%	41.9%	9.6%	4
ASA score 1–2 (vs. 3–4)	5727 (73.3%)	63.4% (3.45–100)	50.7%	77.4%	26.8%	464
BMI, kg/m^2^ [mean (SD)]	24.7 (4.52)	24.9 (22.5–29)	24.2	25.8	1.6	1359
Previous thoracic or abdominal surgery						
No/minor	5757 (73.6%)	91.7% (0–97.8)	84.4%	93.0%	8.6%	1651
Medium/major	410 (5.2%)	8.2% (0–21.1)	6.2%	11.6%	5.4%	
Comorbidities						
Cardiovascular	624 (8%)	7.4% (0–51.6)	4.9%	14.2%	9.2%	0
Respiratory	135 (1.7%)	0% (0–18.8)	0.0%	0.0%	0.0%	
Renal	37 (0.5%)	0% (0–4.2)	0.0%	0.0%	0.0%	
Endocrinological	596 (7.6%)	6.4% (0–37.5)	3.1%	8.7%	5.6%	
Oncological	372 (4.8%)	3.9% (0–37.9)	0.8%	7.7%	6.9%	
Multiple	1347 (17.2%)	19.8% (0–64)	10.3%	28.9%	18.7%	
Number of comorbidities						
None	4515 (57.8%)	51.5% (14–80.0)	37.8%	62.1%	24.3%	0
One	1956 (25%)	25.4% (2.9–67.7)	20.4%	36.4%	16.0%	
Multiple	1347 (17.2%)	19.8% (0–64)	10.3%	28.9%	18.7%	
Tumor location						
Cardia and EGJ	695 (8.9%)	12.4% (0–58.3)	3.7%	19.5%	15.8%	233
Corpus and fundus	3422 (43.8%)	38.2% (18.2–70.6)	29.9%	49.6%	19.8%	
Antrum and pylorus	3039 (38.9%)	40.7% (0–77.8)	30.9%	50.8%	19.9%	
Whole stomach	429 (5.5%	0.4% (0–14.1)	0.0%	2.9%	2.9%	
Tumor category						
cT1–2	1978 (25.3%)	28.2% (0–64.6)	22.5%	37.5%	15.1%	2644
cT3-4	2972 (38.0%	61.7% (0–82)	53.1%	70.3%	17.2%	
cTx	224 (2.9%)	3.1% (0–17.7)	0.0%	10.2%	10.2%	
Nodal category						
cN0	2506 (32.1%)	37.6% (0–77.4)	30.1%	53.1%	23.0%	2640
cN1–3	2275 (29.1%)	48.4% (0–83.3)	36.8%	58.9%	22.1%	
cNx	397 (5.1%)	3.9% (0–59.8)	0.0%	14.7%	14.7%	
Neoadjuvant chemotherapy	1983 (25.4%)	45.2% (4.3–96)	31.3%	66.7%	35.3%	737
Total gastrectomy (vs. subtotal)	2924 (37.4%)	48.0% (22.3–91.7)	35.4%	62.0%	26.6%	0
Open surgery (vs. MI)	3402 (43.5%)	74.2% (12.9–100)	39.0%	92.4%	53.4%	0
Multivisceral resection	1448 (18.5%)	15.9% (0–96.6)	10.9%	25.0%	14.1%	0
Lymph node dissection						
D1 (+)	2578 (33.0%)	15.5% (0–95.2)	4.1%	29.5%	25.4%	0
D2 (+)	5194 (66.4%)	84.2% (4.8–100)	66.9%	94.1%	26.0%	
D3	46 (0.6%)	0% (0–8.1)	0.0%	0.0%	0.0%	

ASA: American society of anesthesiologists, SD: Standard deviation, EGJ: Esophagogastric junction, MI: Minimally invasive (either laparoscopic or robotic)

Neoadjuvant chemotherapy was administered in 4.3% up to 96% (IQR: 35.3%) of the patients across the hospitals and the rate of patients undergoing a total gastrectomy (versus subtotal) ranged from 22.3 to 91.7% (IQR: 26.6%). The proportion of patients undergoing a D2 lymph node dissection ranged per hospital from 4.8 to 100% of the patients (IQR: 26.0%), with a D1(+) lymph node dissection being performed in 0–95.2% of patients (IQR: 25.4%).

### Hospital variation

The overall 30-day mortality rate was 1.2% and rate of severe complications (≥ CD 3a) was 12.1%, with substantial variation in surgical outcomes across hospitals (Table 2). Observed 30-day mortality and severe complications ranged from 0 to 9.7% (IQR: 3.2%) and 5.3–17.9% (IQR: 7.7%). Escalation of care was seen in 4.4–10.4% (IQR: 7.7%). Larger variation between hospitals was observed for retrieval of > 15 lymph nodes (IQR: 12.3%, range 33.3–100%), prolonged hospitalization (IQR: 14.4%, range 0−66.7%) and readmissions (IQR 11.3%, range 0–25%).

**Table 2:** Surgical outcomes

Variable	Denominator	Total	Per hospital				Missing
		%	Median (%) (range)	Q1 (%)	Q3 (%)	IQR (%)	
> 15 LN retrieved	7362	88.1	91.5 (33.3–100)	81.9	94.1	12.3	456
Negative margin (R0)	7817	95.7	93.8 (74.1–100)	90.6	91.0	6.4	1
Severe complications (≥ CD 3a)	7818	12.1	14.5 (5.3–31)	10.3	17.9	7.7	0
Escalation of care^a^	7546	4.2	8.1 (0–17.8)	4.4	10.4	6.0	272
Reoperation^b^	5264	4.8	9.4 (0–27.6)	5.5	12.9	7.5	2554
Proportion > 14 DoH	7562	19.2	21.0 (0–66.7)	14.%	26.9	14.4	256
Readmission (30-day)	4990	5.6	11.8 (0–25)	4.6	15.9	11.3	2828
Mortality (30-day)	7818	1.2	1.94 (0–9.7)	0.0	3.2	3.2	0

LN: Lymph nodes, DoH: Days of hospitalization, CD: Clavien-Dindo

Values are percentages, unless otherwise indicated.

^a^ Unplanned readmission to a higher surveillance unit (either intermediate- or intensive care unit).

^b^ Surgical intervention under general anesthesia.

The crude models reduced hospital variation. Adjusting for case-mix and treatment-related factors did not result in statistically significant changes in the hospital-specific estimated probabilities for any outcome, compared to the crude estimates (Figs. 1 and 2 and Supplementary Figs. 2–7).

**Fig. 1:**
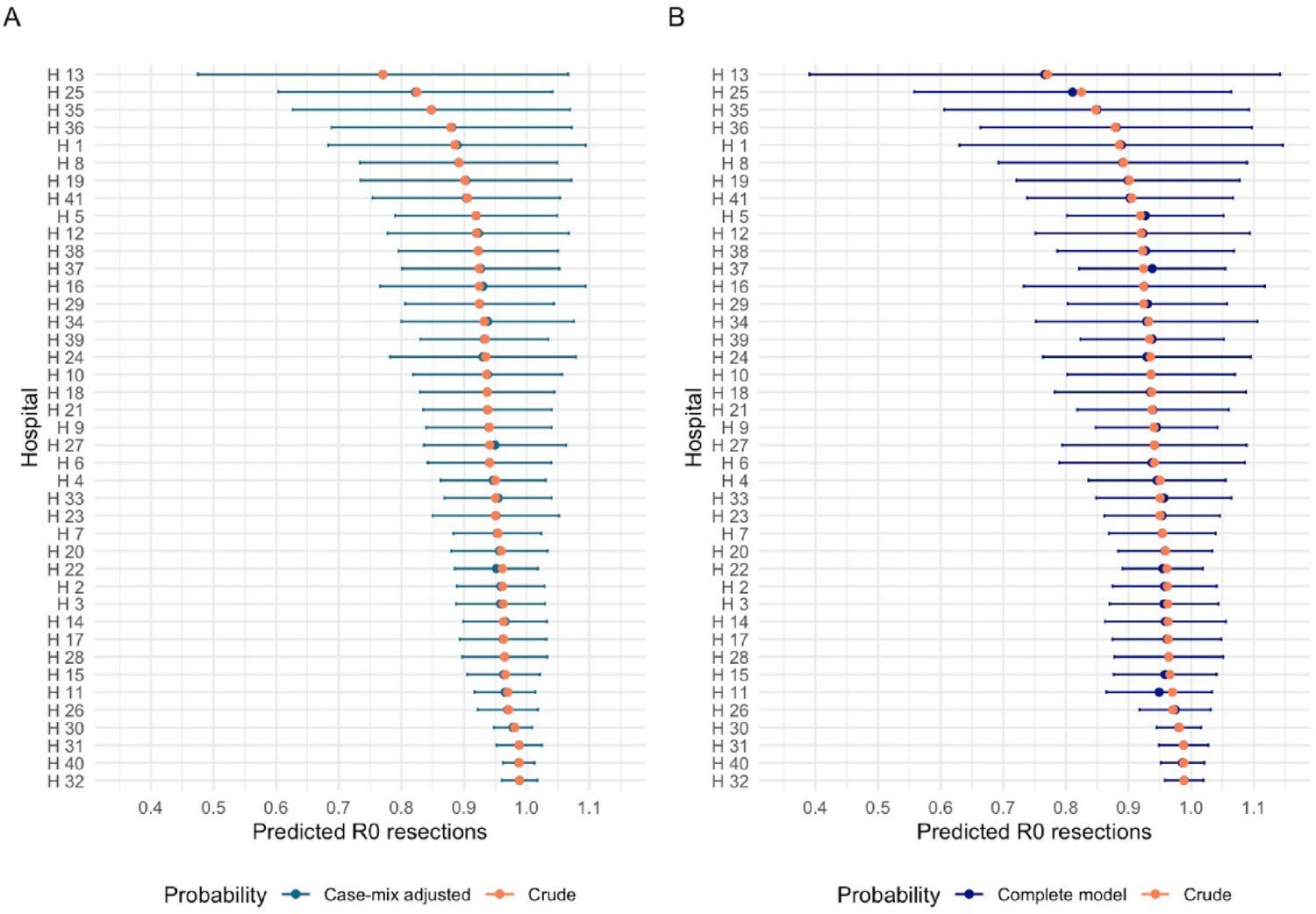
**A**, **B** Estimated probabilities of negative resection margins per hospital (**A**) Crude vs. adjustment for case-mix (**B**) Crude vs. adjustment for case-mix and treatment-related factors (*n* = 7817)

**Fig. 2:**
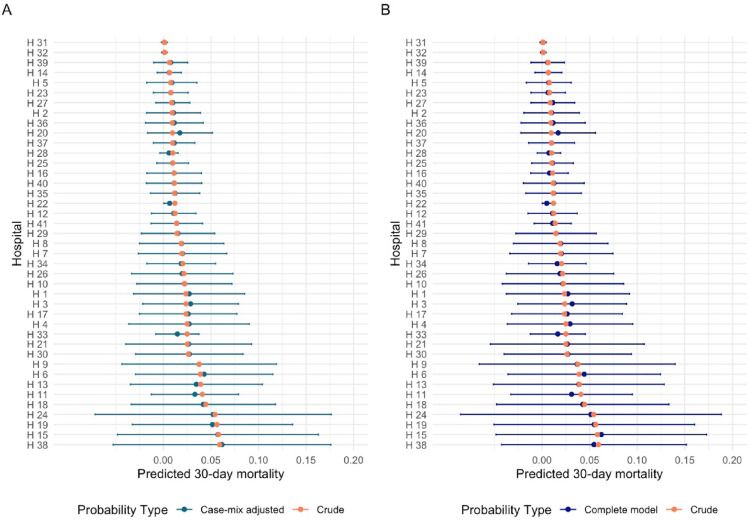
**A**, **B** Estimated probabilities of 30-day mortality per hospital (**A**) Crude vs. adjustment for case-mix (**B**) Crude vs. adjustment for case-mix and treatment-related factors (*n* = 7818)

### Explained variance - Pseudo-R^2^

**Fig. 3:**
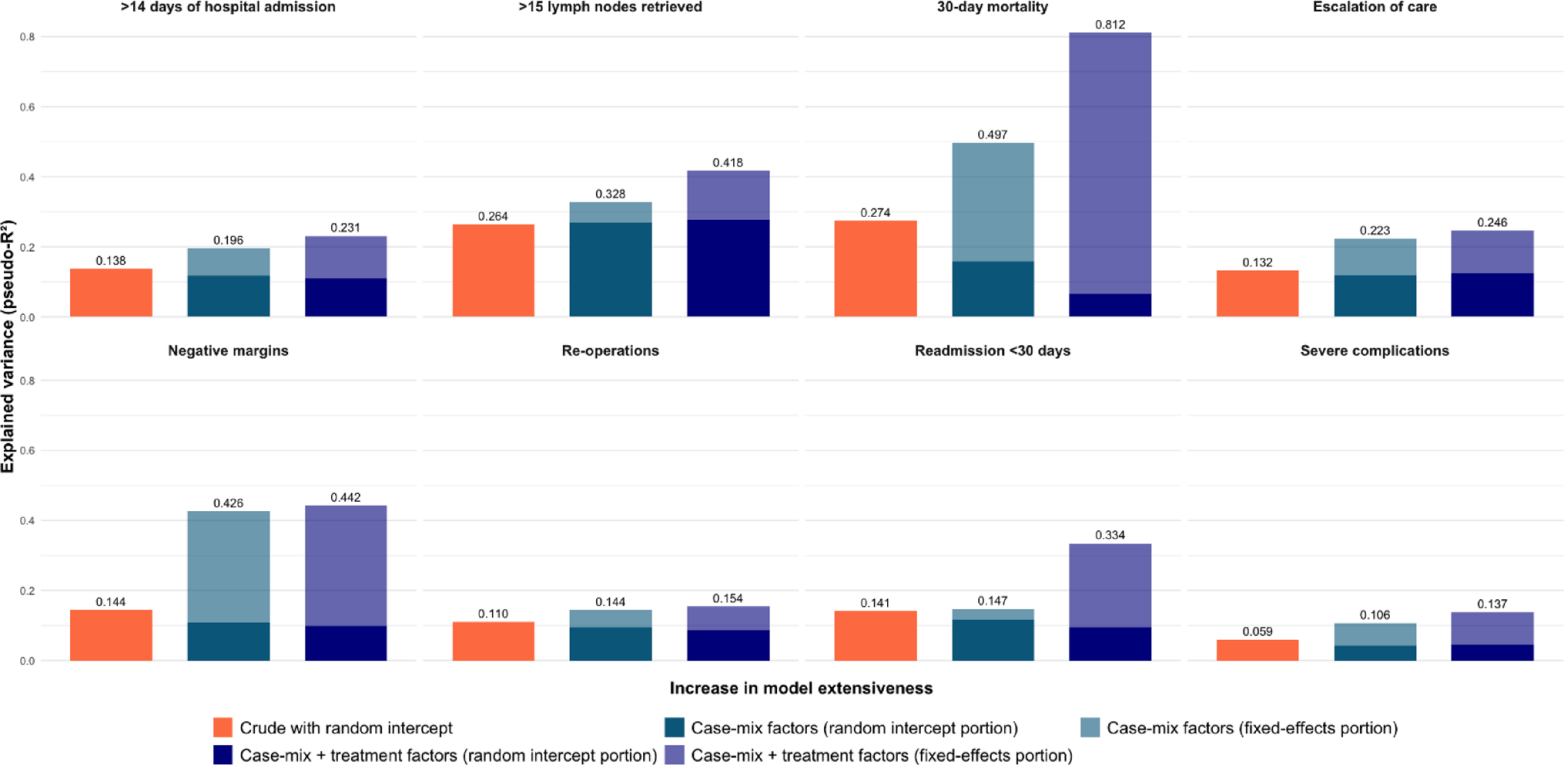
The proportion of explained variance by each of the models according to the conditional pseudo-R^2^. A score of 1 is equal to 100% variance explained

Case-mix factors explained less than 10% of the variance for all outcomes, except for 30-day mortality (33.9%) and negative resection margins (31.7%) (Fig. 3 and supplementary Table 1) as determined by the marginal pseudo-R^2^. Adding treatment-related factors to the models increased the explained variance for 30-day mortality by 40.8% and for readmissions by 20.9%, but had a low to modest impact (< 10%) on the variance of other surgical outcomes. The only model that explained more than half of the variance was the final model for 30-day mortality, including both case-mix and treatment factors. In all other models, the majority of variance remained unexplained.

The probability of treatment in each hospital based on case-mix characteristics was different for the two large Asian centers compared to the other hospitals (Supplementary Fig. 8). However, in the consequently performed sensitivity analyses, excluding patients from the two large Asian centers did not alter findings, except for 30-day mortality (Supplementary Table 2). The marginal pseudo-R^2^ for case-mix factors increased to 47.4% (increase of 13.5%), but decreased for treatment factors to 18.3% (decrease of 22.5%).

## Discussion

In this study, we evaluated the impact of case-mix and treatment-related factors on variation in surgical outcomes following an oncological gastrectomy across 41 hospitals. There was substantial variation in surgical outcomes and adjustment for case-mix and treatment-related factors did not change the estimated hospital outcomes. Case-mix characteristics contributed to the explained variance for 30-day mortality and negative resection margins, but its contribution was low to moderate for other surgical outcomes and for the majority of the outcomes the random intercept contributed the most to the explained variance, indicating that some of the variation reflects statistical uncertainty. These results indicate that case-mix and treatment-related factors explain variation in outcomes to a limited extent, showing where risk adjustment is applicable and where caution is warranted.

Except for the model for 30-day mortality, the models including the case-mix and treatment factors performed poorly for the other outcomes. While the most comprehensive model explained most of the observed variance in 30-day mortality (81.2%), it did not significantly change estimated probabilities for any hospital, likely due to case-mix characteristics balancing each other out within each hospital. Moreover, this reflects a limitation of interpreting model fit statistics as proxies for real-world benchmarking impact. These findings align with a recent study that developed case-mix adjustment models for gastric cancer outcomes for the national Upper-GI registry in the Netherlands (DUCA) [15]. They reported that case-mix had limited effect on inter-hospital differences in outcomes besides 30-day mortality, implicating that extensive case-mix adjustment might unnecessarily complicate comparisons without improving validity for the other outcomes. Taken together, these results suggest that case-mix and treatment adjustment can improve the validity of comparisons for 30-day mortality, but is of limited use for comparing other investigated outcomes.

Unmeasured factors and statistical uncertainty probably contribute to a large extent in the observed variation between hospitals, underlined by the proportion of variance explained by the random intercept for the majority of the outcomes. Surgical proficiency, technical modifications and hospital-specific organization, such as the presence of specialized multidisciplinary teams and clinical care pathways, are often not documented but have a great impact on surgical outcomes. Technical proficiency of a surgeon has been associated with fewer complications, reoperations, readmissions and lower mortality for several surgical procedures, including other upper-GI procedures such as esophagectomy and bariatric surgery [16–18]. In 2016, a questionnaire demonstrated there are substantial differences in clinical pathways for oesophagogastric surgery across European centers [19]. The role and functionality of multidisciplinary teams, the support of clinical nurse specialists and the possibility of nutritional intervention by dietitians varied strongly across the 10 investigated countries, often due to resource constraints. The benefit of these clinical pathways has been established previously. A Cochrane meta-analysis of over 11.000 patients treated in randomized clinical trials found that implementation of clinical pathways was associated with reduced length of hospital admission and lower in-hospital complication rates, reducing healthcare costs [20, 21]. Moreover, high adherence to care pathways, most notably the ERAS protocol following gastric cancer surgery, is associated with improved outcomes, including reduced morbidity, lower rates of readmissions and shorter hospital admission [22–24].

A second notion from our findings is that the investigated outcomes may be inappropriate to compare and rank hospitals as the median case load was only 81 patients with low event rates for several outcomes. Given the typical annual caseload for European centers being less than 100 patients, accurately estimating true outcomes becomes challenging due to a low number of events, which increases the susceptibility to random variation and registration bias [25, 26]. This undermines the reliability of an outcome indicator, resulting in poor ‘rankability’. Previous studies have shown that for multiple oncological resections, the reliability of ranking with surgical outcome indicators is poor and most of the observed variation is attributable to chance [27]. This may also hold for gastric cancer outcomes, but further research is needed to establish this. Composite measures such as *textbook outcome* and *failure to cure*, have been proposed to improve the rankability of hospitals [28, 29]. These measures combine multiple quality metrics which can result in better reliability for hospital comparisons, but they lack specific information on areas requiring improvement [30, 31]. Hence, the development of an optimal benchmarking framework remains pending.

To our knowledge, this is the first study to investigate the role of case-mix and treatment related factors in global hospital variation following an oncological gastrectomy. Our dataset allowed assessment of its impact on the variation in surgical outcomes across different continents. However, our study has several limitations. Despite using extensive models to estimate outcome probabilities per hospital, residual confounding is a potential issue, as not all case-mix factors influencing outcomes may have been measured or adjusted for. In particular, specific comorbidities can affect some of the investigated outcomes [32]. However, we assessed whether individual comorbidities improved model performance based on the AIC, but as this was not the case we opted to include the number of comorbidities as variable instead. Additionally, preoperative weight loss, serving as surrogate for a patients’ metabolic state, have been shown to influence 30-day mortality [32, 33]. However, data on nutritional status or weight loss was not included in the dataset. Furthermore, we only included patients from centers with dedicated upper-GI teams. This restriction may have influenced the results as these centers are often better equipped to handle complex cases and have more robust quality control measures, also illustrated by the low mortality and severe complication rates of 1.2% and 12.1%, respectively. This could limit the effect of case-mix on outcomes as these centers may adequately tailor treatment based on case-mix characteristics and are better able to mitigate surgical risks. On the other hand, these centers are more likely to manage a diverse range of patients, including highly complex cases. Lastly, we did have data on surgical methods used for type of reconstruction and technique of anastomosis, but approximately 60% was missing with the missing data pattern being non-random. Hence, multiple imputation was not feasible and these variables could not be included as treatment factor.

In conclusion, this study highlights that case mix and treatment-related factors do not explain variation observed between hospitals to a large extent. The low contribution of case-mix to explained variance, the substantial impact of unmeasured effects, and the absence of significant changes in the estimated probabilities after adjustment indicate that case-mix and treatment characteristics are not the primary drivers of hospital variation. However, case-mix and treatment characteristics do seem essential for outcomes directly related to survival and oncologic adequacy (i.e. 30-day mortality and R0 resection). Rather than a limitation, the limited explanatory power of case-mix factors for other outcomes highlights the transparency of benchmarking efforts—showing where risk adjustment is informative and where caution is warranted. Additionally, for global comparisons of gastric cancer outcomes, accounting for statistical uncertainty with a random intercept is recommended.

## Supplementary Information

Below is the link to the electronic supplementary material.

Supplementary Material 1

## Data Availability

The datasets used and analysed will be available upon reasonable request.

## References

[1] Sung H, Ferlay J, Siegel RL, Laversanne M, Soerjomataram I, Jemal A, et al. Global cancer statistics 2020: GLOBOCAN estimates of incidence and mortality worldwide for 36 cancers in 185 countries. CA Cancer J Clin. 2021;71(3):209–49. 10.3322/caac.21660. 33538338 10.3322/caac.21660

[2] Brenkman HJF, Claassen L, Hannink G, van der Werf LR, Ruurda JP, Nieuwenhuizen GAP, et al. Learning curve of laparoscopic gastrectomy: a multicenter study. Ann Surg. 2023;277(4):e808–16. 10.1097/SLA.0000000000005479. 35801714 10.1097/SLA.0000000000005479

[3] Guller U, Warschkow R, Ackermann CJ, Schmied B, Cerny T, Ess S. Lower hospital volume is associated with higher mortality after oesophageal, gastric, pancreatic and rectal cancer resection. Swiss Med Wkly. 2017;147:w14473. 10.4414/smw.2017.14473. 28750418 10.4414/smw.2017.14473

[4] Breuer E, Mueller M, Doyle MB, Yang L, Darwish Murad S, Anwar IJ, et al. Liver transplantation as a new standard of care in patients with perihilar cholangiocarcinoma? Results from an international benchmark study. Ann Surg. 2022;276(5):846–53. 10.1097/SLA.0000000000005641. 35894433 10.1097/SLA.0000000000005641 PMC9983747

[5] Gero D, Raptis DA, Vleeschouwers W, van Veldhuisen SL, Martin AS, Xiao Y, et al. Defining global benchmarks in bariatric surgery: a retrospective multicenter analysis of minimally invasive Roux-en-Y gastric bypass and sleeve gastrectomy. Ann Surg. 2019;270(5):859–67. 10.1097/SLA.0000000000003512. 31592894 10.1097/SLA.0000000000003512

[6] Schmidt HM, Gisbertz SS, Moons J, Rouvelas I, Kauppi J, Brown A, et al. Defining benchmarks for transthoracic esophagectomy: a multicenter analysis of total minimally invasive esophagectomy in low risk patients. Ann Surg. 2017;266(5):814–21. 10.1097/SLA.0000000000002445. 28796646 10.1097/SLA.0000000000002445

[7] Staiger RD, Rossler F, Kim MJ, Brown C, Trenti L, Sasaki T, et al. Benchmarks in colorectal surgery: multinational study to define quality thresholds in high and low anterior resection. Br J Surg. 2022;109(12):1274–81. 10.1093/bjs/znac300. 36074702 10.1093/bjs/znac300

[8] Schneider MA, Kim J, Berlth F, Sugita Y, Grimminger PP, Sano T, et al. Defining benchmarks for total and distal gastrectomy: global multicentre analysis. Br J Surg. 2024;111(2):379. 10.1093/bjs/znad379. 10.1093/bjs/znad379 PMC10878554 38377359

[9] Leeftink G, Hans EW. Case mix classification and a benchmark set for surgery scheduling. J Sched. 2018;21(1):17–33. 10.1007/s10951-017-0539-8.

[10] Collaborators GUHD. The burden of stomach cancer mortality by county, race, and ethnicity in the USA, 2000-2019: a systematic analysis of health disparities. Lancet Reg Health Am. 2023;24:100547. 10.1016/j.lana.2023.100547. 37600165 10.1016/j.lana.2023.100547 PMC10435837

[11] Abengozar R, Sharma A, Sharma R. Gastric cancer: lessons learned from high-incidence geographic regions. J Gastrointest Oncol. 2021;12(Suppl 2):S350–60. 10.21037/jgo-2019-gi-05. 34422399 10.21037/jgo-2019-gi-05 PMC8343089

[12] Sullivan TR, Lee KJ, Ryan P, Salter AB. Multiple imputation for handling missing outcome data when estimating the relative risk. BMC Med Res Methodol. 2017;17(1):134. 10.1186/s12874-017-0414-5. 28877666 10.1186/s12874-017-0414-5 PMC5588607

[13] Nakagawa S, Johnson PCD, Schielzeth H. The coefficient of determination R(2) and intra-class correlation coefficient from generalized linear mixed-effects models revisited and expanded. J R Soc Interface. 2017. 10.1098/rsif.2017.0213. 28904005 10.1098/rsif.2017.0213 PMC5636267

[14] Austin PC. An introduction to propensity score methods for reducing the effects of confounding in observational studies. Multivariate Behav Res. 2011;46(3):399–424. 10.1080/00273171.2011.568786. 21818162 10.1080/00273171.2011.568786 PMC3144483

[15] van der Linde M, Visser MR, Eijkenaar F, Oude Voshaar MAH, van Hillegersberg R, van Sandick JW, et al. Case-mix adjustment for between-hospital comparisons in oesophageal and gastric cancer surgery. Eur J Surg Oncol. 2025. 10.1016/j.ejso.2025.109644. 40014956 10.1016/j.ejso.2025.109644

[16] Stulberg JJ, Huang R, Kreutzer L, Ban K, Champagne BJ, Steele SR, et al. Association between surgeon technical skills and patient outcomes. JAMA Surg. 2020;155(10):960–8. 10.1001/jamasurg.2020.3007. 32838425 10.1001/jamasurg.2020.3007 PMC7439214

[17] Birkmeyer JD, Finks JF, O’Reilly A, Oerline M, Carlin AM, Nunn AR, et al. Surgical skill and complication rates after bariatric surgery. N Engl J Med. 2013;369(15):1434–42. 10.1056/NEJMsa1300625. 24106936 10.1056/NEJMsa1300625

[18] Ketel MHM, Klarenbeek BR, Abma I, Belgers EHJ, Coene PLO, Dekker JWT, et al. Nationwide association of surgical performance of minimally invasive esophagectomy with patient outcomes. JAMA Netw Open. 2024;7(4):e246556. 10.1001/jamanetworkopen.2024.6556. 38639938 10.1001/jamanetworkopen.2024.6556 PMC11031683

[19] Messager M, de Steur W, Boelens PG, Jensen LS, Mariette C, Reynolds JV, et al. Description and analysis of clinical pathways for oesophago-gastric adenocarcinoma, in 10 European countries (the EURECCA upper gastro intestinal group–European registration of cancer care). Eur J Surg Oncol. 2016;42(9):1432–47. 10.1016/j.ejso.2016.01.001. 26898839 10.1016/j.ejso.2016.01.001

[20] Rotter T, Kinsman L, James E, Machotta A, Gothe H, Willis J, et al. Clinical pathways: effects on professional practice, patient outcomes, length of stay and hospital costs. Cochrane Database Syst Rev. 2010;3:CD006632. 10.1002/14651858.CD006632. 10.1002/14651858.CD006632.pub2 20238347

[21] Deneckere S, Euwema M, Van Herck P, Lodewijckx C, Panella M, Sermeus W, et al. Care pathways lead to better teamwork: results of a systematic review. Soc Sci Med. 2012;75(2):264–8. 10.1016/j.socscimed.2012.02.060. 22560883 10.1016/j.socscimed.2012.02.060

[22] Pisarska M, Pedziwiatr M, Malczak P, Major P, Ochenduszko S, Zub-Pokrowiecka A, et al. Do we really need the full compliance with ERAS protocol in laparoscopic colorectal surgery? A prospective cohort study. Int J Surg. 2016;36(Pt A):377–82. 10.1016/j.ijsu.2016.11.088. 27876677 10.1016/j.ijsu.2016.11.088

[23] Chan DS, Reid TD, White C, Willicombe A, Blackshaw G, Clark GW, et al. Influence of a regional centralised upper gastrointestinal cancer service model on patient safety, quality of care and survival. Clin Oncol (R Coll Radiol). 2013;25(12):719–25. 10.1016/j.clon.2013.08.005. 23994038 10.1016/j.clon.2013.08.005

[24] Wee IJY, Syn NL, Shabbir A, Kim G, So JBY. Enhanced recovery versus conventional care in gastric cancer surgery: a meta-analysis of randomized and non-randomized controlled trials. Gastric Cancer. 2019;22(3):423–34. 10.1007/s10120-019-00937-9. 30805742 10.1007/s10120-019-00937-9

[25] van Dishoeck AM, Lingsma HF, Mackenbach JP, Steyerberg EW. Random variation and rankability of hospitals using outcome indicators. BMJ Qual Saf. 2011;20(10):869–74. 10.1136/bmjqs.2010.048058. 21642443 10.1136/bmjqs.2010.048058

[26] Fischer C, Lingsma HF, van Leersum N, Tollenaar RA, Wouters MW, Steyerberg EW. Comparing colon cancer outcomes: the impact of low hospital case volume and case-mix adjustment. Eur J Surg Oncol. 2015;41(8):1045–53. 10.1016/j.ejso.2015.04.009. 26067372 10.1016/j.ejso.2015.04.009

[27] Henneman D, van Bommel AC, Snijders A, Snijders HS, Tollenaar RA, Wouters MW, et al. Ranking and rankability of hospital postoperative mortality rates in colorectal cancer surgery. Ann Surg. 2014;259(5):844–9. 10.1097/SLA.0000000000000561. 24717374 10.1097/SLA.0000000000000561

[28] Busweiler LA, Schouwenburg MG, van Berge Henegouwen MI, Kolfschoten NE, de Jong PC, Rozema T, et al. Textbook outcome as a composite measure in oesophagogastric cancer surgery. Br J Surg. 2017;104(6):742–50. 10.1002/bjs.10486. 28240357 10.1002/bjs.10486

[29] Voeten DM, van der Werf LR, Wilschut JA, Busweiler LAD, van Sandick JW, van Hillegersberg R, et al. Failure to cure in patients undergoing surgery for gastric cancer: a nationwide cohort study. Ann Surg Oncol. 2021;28(8):4484–96. 10.1245/s10434-020-09510-6. 33486644 10.1245/s10434-020-09510-6 PMC8253712

[30] Hofstede SN, Ceyisakar IE, Lingsma HF, Kringos DS, van de Marang- Mheen PJ. Ranking hospitals: do we gain reliability by using composite rather than individual indicators? BMJ Qual Saf. 2019;28(2):94–102. 10.1136/bmjqs-2017-007669. 29789406 10.1136/bmjqs-2017-007669

[31] Austin PC, Ceyisakar IE, Steyerberg EW, Lingsma HF, van de Marang- Mheen PJ. Ranking hospital performance based on individual indicators: can we increase reliability by creating composite indicators? BMC Med Res Methodol. 2019;19(1):131. 10.1186/s12874-019-0769-x. 31242857 10.1186/s12874-019-0769-x PMC6595591

[32] van der Linde M, Visser MR, Eijkenaar F, Oude Voshaar MAH, van Hillegersberg R, van Sandick JW, et al. Case-mix adjustment for between-hospital comparisons in oesophageal and gastric cancer surgery. Eur J Surg Oncol. 2025;51(6):109644. 10.1016/j.ejso.2025.109644. 40014956 10.1016/j.ejso.2025.109644

[33] Yang Y, Gao P, Song Y, Sun J, Chen X, Zhao J, et al. The prognostic nutritional index is a predictive indicator of prognosis and postoperative complications in gastric cancer: a meta-analysis. Eur J Surg Oncol. 2016;42(8):1176–82. 10.1016/j.ejso.2016.05.029. 27293109 10.1016/j.ejso.2016.05.029

