# Supplementary material for: Hospital variation in surgical outcomes for gastric cancer: the impact of case-mix and treatment across a global cohort
Source: Gastric Cancer. 2026 Jan 13;29(2):424–32. doi: 10.1007/s10120-025-01696-6 (PMC12957621; doi:10.1007/s10120-025-01696-6)
Supplement: Supplementary file 1 — Supplementary Material 1 [file 10120_2025_1696_MOESM1_ESM.docx]

**Supplement**

Sander JM van Hootegem^1^, Margrietha van der Linde^2^, Marcel A Schneider^3^, Jeesun Kim^4^, Felix Berlth^5,6^, Yutaka Sugita^7^, Peter P Grimminger^5^, Gian Luca Baiocchi^8^, Giovanni De Manzoni^9^, Maria Bencivenga^9^, Suzanne Gisbertz^10^, Souya Nunobe^7^, Han-Kwang Yang^4^, Christian A Gutschow^3^, Sjoerd M Lagarde^1^, Hester F Lingsma^2^, Bas PL Wijnhoven^1^, The GastroBenchmark & GASTRODATA consortiums†

^1^ Department of Surgery, Erasmus Medical Center, Rotterdam, The Netherlands

^2^ Department of Public Health, Erasmus Medical Center, Rotterdam, The Netherlands

^3^ Department of Surgery & Transplantation, University Hospital Zürich, Zürich, Switzerland

^4^ Department of Surgery, Seoul National University Cancer Hospital, Seoul, South Korea

^5^ Department of General-, Visceral- and Transplant Surgery, University Medical Center Mainz, Mainz, Germany

^6^ Department of Surgery, University Hospital of Tübingen, Tübingen, Germany

^7^ Department of Gastroenterological Surgery, Cancer Institute Hospital of the Japanese Foundation for Cancer Research, Tokyo, Japan

^8^ Department of Clinical and Experimental Sciences, University of Brescia, Brescia, Italy

^9^ Department of Surgery, University Hospital of Verona, Verona, Italy

^10^ Department of Surgery, Amsterdam UMC location University of Amsterdam, Amsterdam, The Netherlands

^11^ Cancer Treatment and Quality of Life, Cancer Centre Amsterdam, Amsterdam, The Netherlands

† Listed under the header Collaborators in the supplement.

**Corresponding author:**

SJM van Hootegem

Department of Surgery, Erasmus MC, University Medical Center

P.O. Box 2040, 3000CA Rotterdam, The Netherlands

**Table of contents**

**Supplement 1.** Collaborators and participating centers

**Supplement 2.** Supplementary figures

**S1.** Collaborators and participating centers

**Collaborators**

*GastroBenchmark Consortium*

Hidde Overtoom (Department of Surgery, Erasmus University Medical Center, Rotterdam, The Netherlands); Ines Gockel (Department of Visceral, Transplant, Thoracic and Vascular Surgery, University Hospital of Leipzig, Leipzig, Germany); René Thieme (Department of Visceral, Transplant, Thoracic and Vascular Surgery, University Hospital of Leipzig, Leipzig, Germany); Ewen A. Griffiths (Department of Upper GI Surgery, Queen Elizabeth Hospital, University Hospitals Birmingham NHS Foundation Trust, Birmingham, UK); William Butterworth (Department of Upper GI Surgery, Queen Elizabeth Hospital, University Hospitals Birmingham NHS Foundation Trust, Birmingham, UK); Henrik Nienhüser (Klinik für Allgemein-, Viszeral- und Transplantationschirurgie, Universitätsklinikum Heidelberg, Heidelberg, Germany); Beat Müller (Klinik für Allgemein-, Viszeral- und Transplantationschirurgie, Universitätsklinikum Heidelberg, Heidelberg, Germany); Nerma Crnovrsanin (Klinik für Allgemein-, Viszeral- und Transplantationschirurgie, Universitätsklinikum Heidelberg, Heidelberg, Germany); Felix Nickel (Department of General, Visceral, and Thoracic Surgery, University Medical Center Hamburg-Eppendorf, Hamburg, Germany); Suzanne S. Gisbertz (Department of Surgery, Amsterdam UMC, University of Amsterdam, & Cancer Center Amsterdam, Cancer Treatment and Quality of Life, Amsterdam, The Netherlands ); Mark I. van Berge Henegouwen (Department of Surgery, Amsterdam UMC, University of Amsterdam, & Cancer Center Amsterdam, Cancer Treatment and Quality of Life, Amsterdam, The Netherlands); Philip H. Pucher (Department of Surgery, Queen Alexandra Hospital, Portsmouth Hospitals NHS Trust, Portsmouth, UK); Kashuf Khan (Department of Surgery, Queen Alexandra Hospital, Portsmouth Hospitals NHS Trust, Portsmouth, UK); Asif Chaudry (The Royal Marsden NHS Foundation Trust, Chelsea, London, SW3 6JJ, UK); Pranav H. Patel (The Royal Marsden NHS Foundation Trust, Chelsea, London, SW3 6JJ, UK); Manuel Pera (Section of Gastrointestinal Surgery, Hospital Universitario del Mar, Universitat Autònoma de Barcelona, Barcelona, Spain); Mariagiulia Dal Cero (Section of Gastrointestinal Surgery, Hospital Universitario del Mar, Universitat Autònoma de Barcelona, Barcelona, Spain); Carlos Garcia (Hospital San Borja Arriarán, Av. Sta. Rosa 1234, Santiago, Región Metropolitana, Chile); Guillermo Martinez Salinas (Hospital San Borja Arriarán, Av. Sta. Rosa 1234, Santiago, Región Metropolitana, Chile); Paulo Kassab (Gastroesophageal and Bariatric Surgical Division, Department of Surgery, Santa Casa of São Paulo Medical School and Hospital, São Paulo, Brazil); Osvaldo Antônio Prado Castro (Gastroesophageal and Bariatric Surgical Division, Department of Surgery, Santa Casa of São Paulo Medical School and Hospital, São Paulo, Brazil); Enrique Norero (Esophagogastric Surgery Unit, Digestive Surgery Department, Hospital Dr Sotero del Rio, Pontificia Universidad Catolica de Chile, Santiago, Chile); Paul Wisniowski (Division of Upper GI and General Surgery, Keck School of Medicine, University of Southern California, 1510 San Pablo St., Health Sciences Campus, Los Angeles, USA); Luke Randall Putnam (Division of Upper GI and General Surgery, Keck School of Medicine, University of Southern California, 1510 San Pablo St., Health Sciences Campus, Los Angeles, USA); Pietro Maria Lombardi (Division of Minimally Invasive Surgical Oncology, Niguarda Cancer Center, ASST Grande Ospedale Metropolitano Niguarda, Piazza Ospedale Maggiore, 3, 20162, Milan, Italy); Giovanni Ferrari (Division of Minimally Invasive Surgical Oncology, Niguarda Cancer Center, ASST Grande Ospedale Metropolitano Niguarda, Piazza Ospedale Maggiore, 3, 20162, Milan, Italy); Rita Gudaityte (Department of Surgery, Hospital of Lithuanian University of Health Sciences, Eiveniu 2, Kaunas 50161, Lithuania); Almantas Maleckas (Department of Surgery, Hospital of Lithuanian University of Health Sciences, Eiveniu 2, Kaunas 50161, Lithuania); Leanne Prodehl (Department of Surgery, Charlotte Maxeke Johannesburg Academic Hospital, University of the Witwatersrand, Johannesburg, South Africa); Antonio Castaldi (Service de Chirurgie Digestive et Cancérologie Digestive, Hôpital Universitaire Carémeau, Nîmes, France); Michel Prudhomme (Service de Chirurgie Digestive et Cancérologie Digestive, Hôpital Universitaire Carémeau, Nîmes, France); Simone Giacopuzzi (Department of Surgery, University Hospital of Verona, Verona, Italy); Riccardo Rosati (Department of Surgery, San Raffaele Hospital, Milano, Italy); Francesco Puccetti (Department of Surgery, San Raffaele Hospital, Milano, Italy); Domenico D'Ugo (FONDAZIONE POLICLINICO UNIVERSITARIO GEMELLI-IRCCS, Roma, Italy); Daniel Gero (Department of Surgery & Transplantation, University Hospital Zürich, Raemistrasse 100, 8091 Zurich, Switzerland); Hyuk-Joon Lee (Department of Surgery, Seoul National University Cancer Hospital, 101 Daehak-ro Jongno-gu, Seoul, South Korea).

*GASTRODATA Consortium*

Guillaume Piessen (Department of Surgery, University Hospital of Lille, Lille, France); Justine Lerooy (Department of Surgery, University Hospital of Lille, Lille, France); Johanna Wilhelmina van Sandick (Department of Surgical Oncology, The Netherlands Cancer Institute—Antoni van Leeuwenhoek Hospital, Amsterdam, The Netherlands); Suzanne S. Gisbertz (Department of Surgery, Amsterdam UMC, University of Amsterdam, & Cancer Center Amsterdam, Cancer Treatment and Quality of Life, Amsterdam, The Netherlands ); Mark I. van Berge Henegouwen (Department of Surgery, Amsterdam UMC, University of Amsterdam, & Cancer Center Amsterdam, Cancer Treatment and Quality of Life, Amsterdam, The Netherlands); Jessie Elliott (Department of Surgery, Trinity St. James's Cancer Institute, St. James's Hospital and Trinity College Dublin, Dublin, Ireland); Paolo Morgagni (GB Morgagni-L Pierantoni Hospital, Forlì, Italy); Arnulf H. Hölscher (Contilia Center for Esophageal Diseases, Elisabeth Hospital Essen, West German Tumor Center, University Medicine Essen, Germany); Martin Hemmerich (Contilia Center for Esophageal Diseases, Elisabeth Hospital Essen, West German Tumor Center, University Medicine Essen, Germany); Stefan Mönig (Department of Surgery, University Hospital of Geneva, Geneva, Switzerland); Mickael Chevallay (Department of Surgery, University Hospital of Geneva, Geneva, Switzerland); Piotr Kołodziejczyk (Department of Surgery, Jagiellonian University, Kraków, Poland); Henk Hartgrink (Leiden University Medical Center, Leiden, The Netherlands); Paulo Matos da Costa (Faculdade de Medicina, Universidade de Lisboa; Lisboa, Portugal); Filipe Castro Borges (Faculdade de Medicina, Universidade de Lisboa; Lisboa, Portugal); Andrew Davies (Department of Surgery, Guy’s & St Thomas’ NHS Foundation Trust, London, UK); Cara Baker (Department of Surgery, Guy’s & St Thomas’ NHS Foundation Trust, London, UK); William Allum (The Royal Marsden NHS Foundation Trust, Chelsea, London, SW3 6JJ, UK); Sacheen Kumar (The Royal Marsden NHS Foundation Trust, Chelsea, London, SW3 6JJ, UK); Wojciech Polkowski (Medical University of Lublin, Lublin, Poland); Karol Rawicz-Pruszyński (Medical University of Lublin, Lublin, Poland); Uberto Fumagalli Romario (Digestive Surgery, European Institute of Oncology, IRCCS, Milano, Italy); Stefano De Pascale (Digestive Surgery, European Institute of Oncology, IRCCS, Milano, Italy); Antonio Tarasconi (Department of Surgery, University Hospital of Brescia, Brescia, Italy); Daniel Reim (Department of Surgery, TUM School of Medicine, Technical University of Munich, Germany); Ilaria Pergolini (Department of Surgery, TUM School of Medicine, Technical University of Munich, Germany); Lucio Lara Santos (Department of Surgery, Portuguese Institute of Oncology, Porto, Portugal); Pedro Carvalho Martins (Department of Surgery, Portuguese Institute of Oncology, Porto, Portugal); Alberto Biondi (FONDAZIONE POLICLINICO UNIVERSITARIO GEMELLI-IRCCS, Roma, Italy); Riccardo Rosati (Department of Surgery, San Raffaele Hospital, Milano, Italy); Maurizio Degiuli (Department of Oncology, University of Turin, San Luigi University Hospital, Turin, Italy); Rossella Reddavid (Department of Oncology, University of Turin, San Luigi University Hospital, Turin, Italy); Wojciech Kielan (University Centre of General and Oncological Surgery, Medical University Wroclaw, Poland); Paul Magnus Schneider (Digestive Oncology Tumor Center and Esophageal Cancer Center, Hirslanden Medical Center, Zurich, Switzerland); Thomas Murphy (Mercy University Hospital, Cork, Ireland).

**Participating centers**

Asia:

- Republic of Korea, Seoul, National University Cancer Hospital
- Japan, Tokyo, Cancer Institute Hospital of the JFCR

Europe:

- France, Nimes, Hôpital Universitaire Carémeau
- Germany, Heidelberg, University Hospital
- Germany, Leipzig, University Hospital
- Germany, Mainz, University Hospital
- Italy, Milano, Niguarda Hospital
- Lithuania, Kaunas, Hospital of Lithuanian University of Health Sciences
- Netherlands, Rotterdam, Erasmus Medical Center
- Spain, Barcelona, Hospital del Mar
- Switzerland, Zurich, University Hospital
- United Kingdom, Birmingham, Queen Elizabeth University Hospital
- United Kingdom, Portsmouth, Queen Alexandra Hospital
- United Kingdom, London, The Royal Marsdon (Prof. A. Chaudry)

South America:

- Brazil, São Paulo, Santa Casa
- Chile, Santiago, Hospital San Borja Arriarán
- Chile, Santiago, Hospital Dr Sotero del Rio, Pontificia Universidad Catolica de Chile

North America:

- USA, Los Angeles/CA, Keck Medical School

Africa:

- South Africa, Johannesburg, Charlotte Maxeke Johannesburg Academic Hospital/ University of the Witwatersrand

GASTRODATA Collaborative (Europe):

- France, Lille, Centre hospitaire universitaire
- Germany, Frankfurt, Agaplesion Markus Hospital
- Germany, Munich, Technical University
- Ireland, Cork, Mercy University Hospital
- Ireland, Dublin, Trinity St. James's Cancer Institute
- Italy, Brescia, University Hospital
- Italy, Forlì, GB Morgagni-L Pierantoni Hospital
- Italy, Milano, Istituto Europeo di Oncologia
- Italy, Milano, San Raffaele Hospital
- Italy, Roma, Cattolica University
- Italy, Torino, University of Torino
- Italy, Verona, University Hospital
- Netherlands, Amsterdam, Amsterdam UMC
- Netherlands, Amsterdam, The Netherlands Cancer Institute
- Netherlands, Leiden, University Medical Center
- Poland, Lublin, Medical University
- Poland, Kraków, Jagiellonian University
- Poland, Wroclaw, Medical University
- Portugal, Lisbon, University of Lisbon
- Portugal, Porto, Portuguese Institute of Oncology
- Switzerland, Geneva, University Hospital
- Switzerland, Zurich, Hirslanden Medical Center
- United Kingdom, London, St. Thomas’ NHS
- United Kingdom, London, The Royal Marsden (Prof. W. Allum)

**S2.** Supplementary figures and tables

**Supplementary figure 1** Study flowchart.


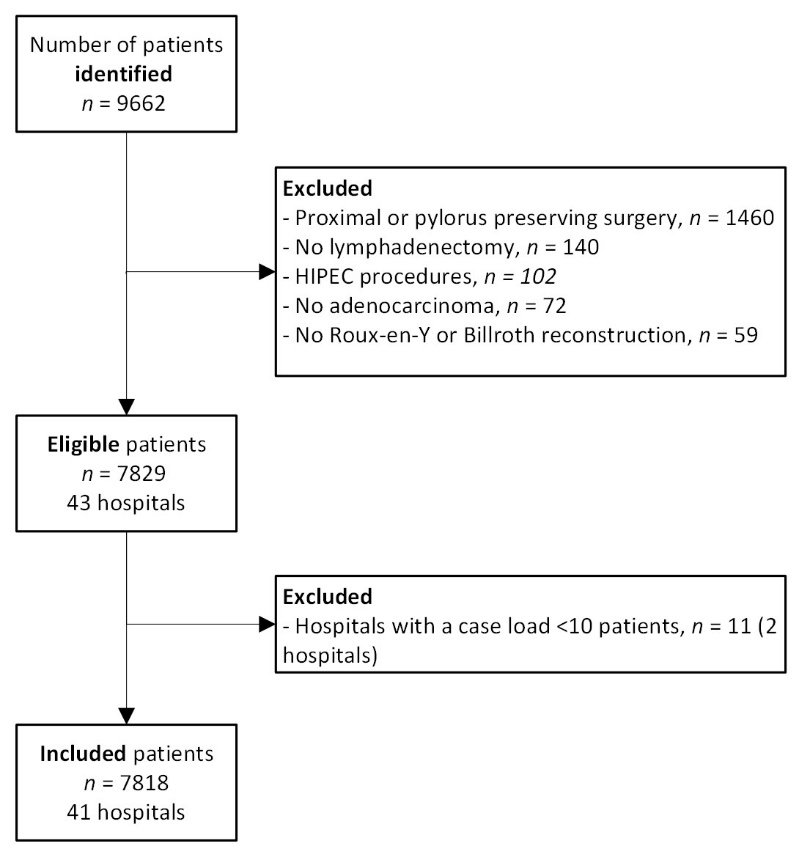


**Supplementary figure 2A-B** Estimated probabilities of >15 lymph nodes retrieved per hospital A. Crude vs. adjustment for case-mix B. Crude vs. adjustment for case-mix and treatment-related factors (*n* = 7362).

**
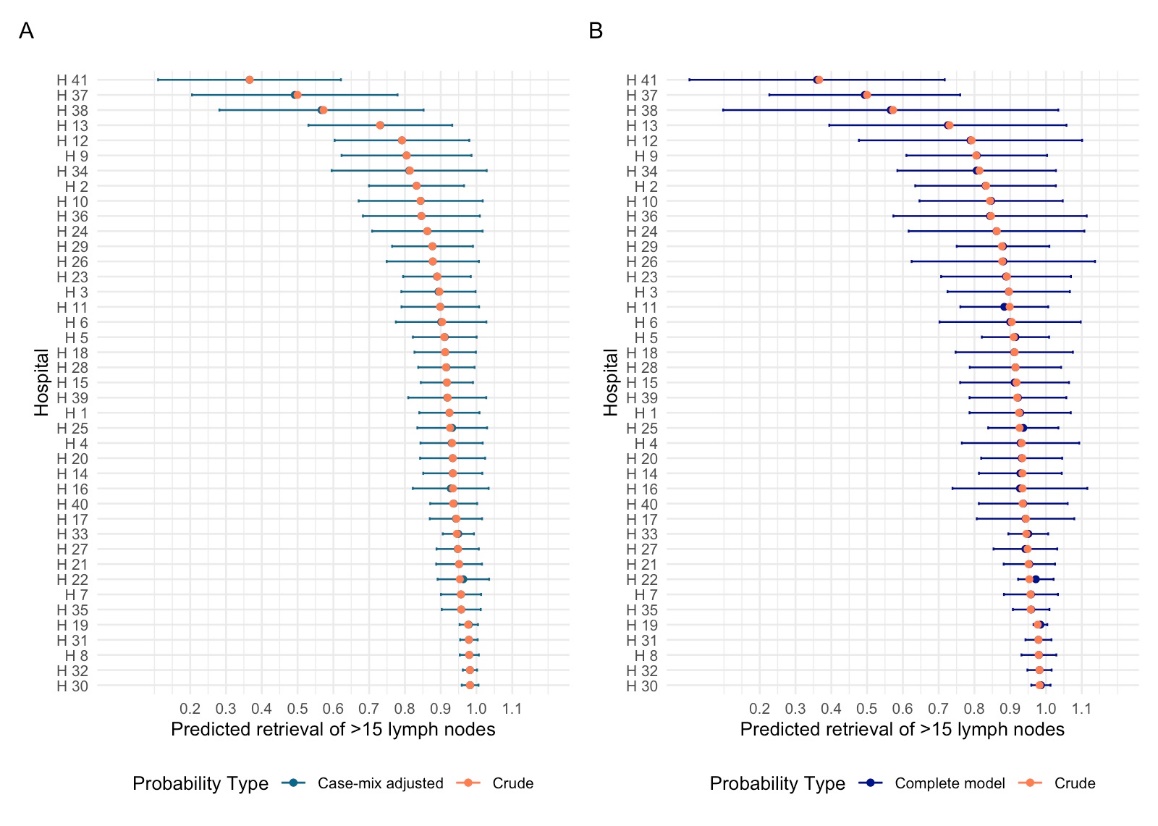
**

**Supplementary figure 3A-B** Estimated probabilities of severe complications per hospital A. Crude vs. adjustment for case-mix B Crude vs. adjustment for case-mix and treatment-related factors (*n* = 7818).

**
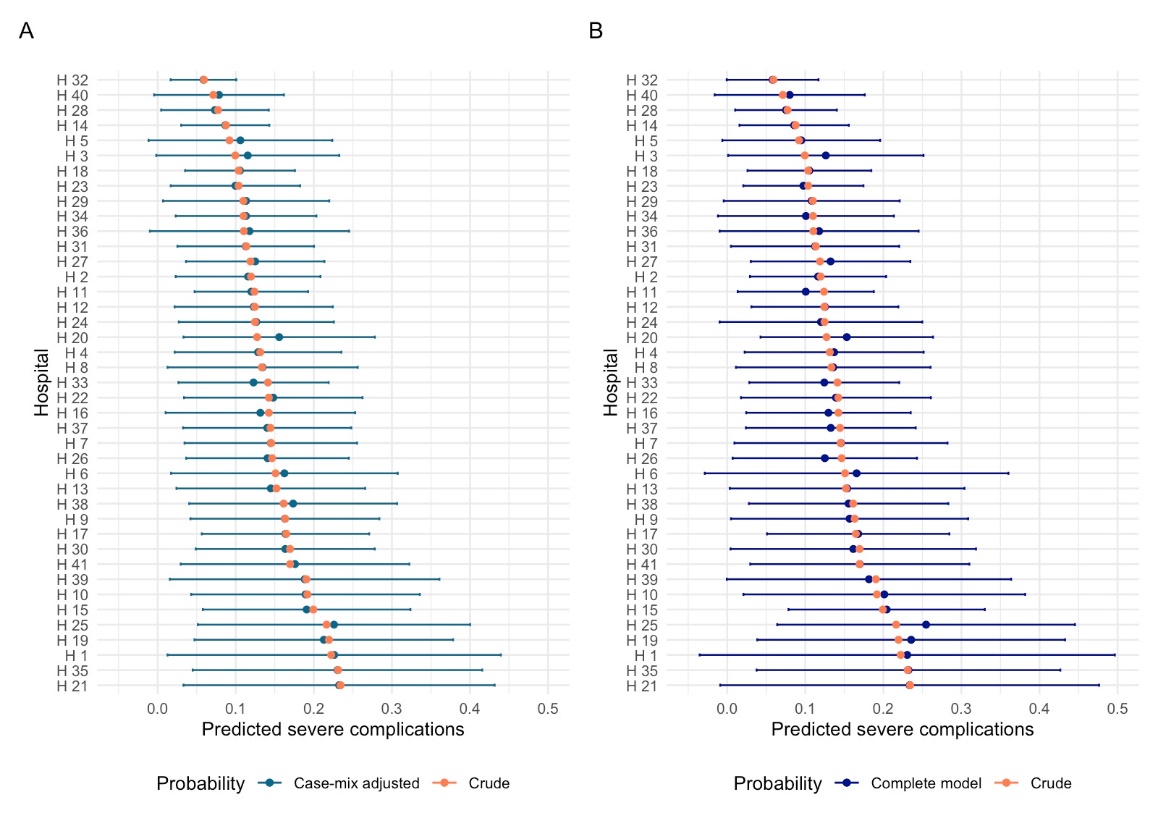
**

**Supplementary figure 4A-B** Estimated probabilities of reoperations per hospital A. Crude vs. adjustment for case-mix B Crude vs. adjustment for case-mix and treatment-related factors (*n* = 5264).

**
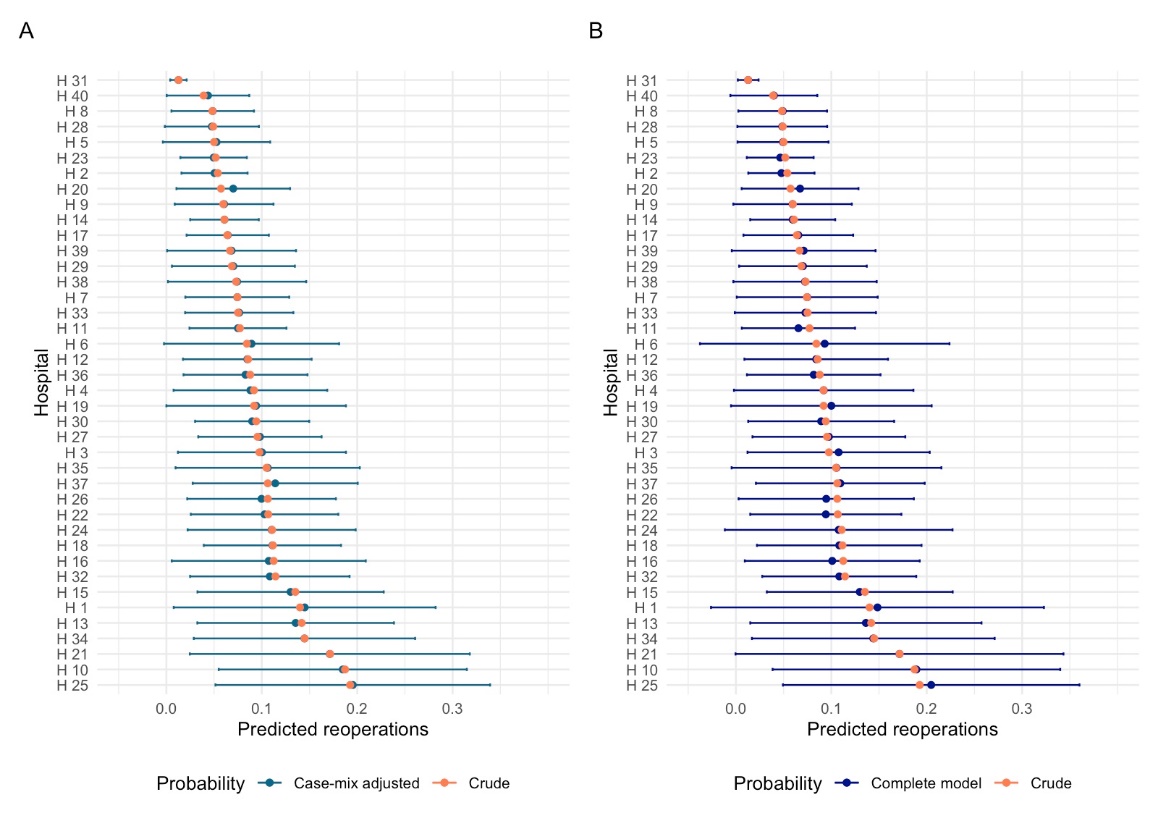
**

**Supplementary figure 5A-B** Estimated probabilities of escalation of care per hospital A. Crude vs. adjustment for case-mix B Crude vs. adjustment for case-mix and treatment-related factors (*n* = 7546).

**
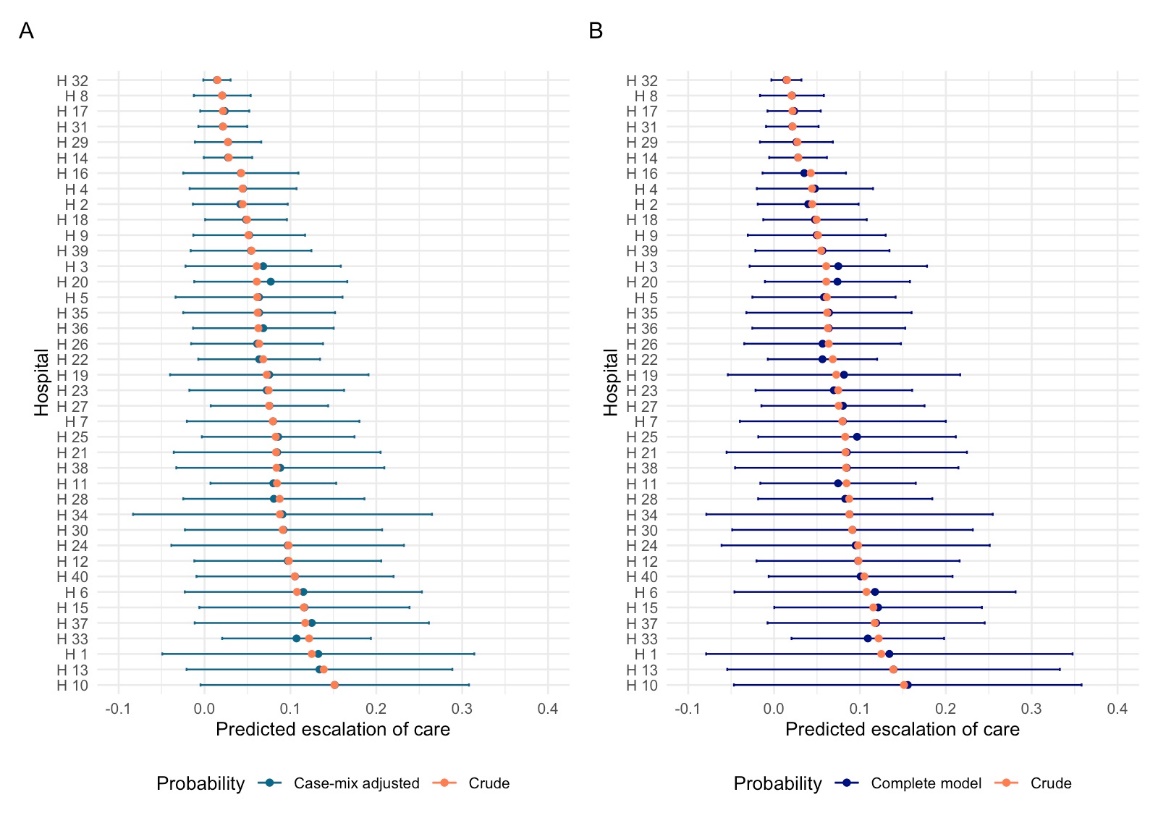
**

**Supplementary figure 6A-B** Estimated probabilities of prolonged hospital stay per hospital A. Crude vs. adjustment for case-mix B Crude vs. adjustment for case-mix and treatment-related factors (*n* = 7562).

**
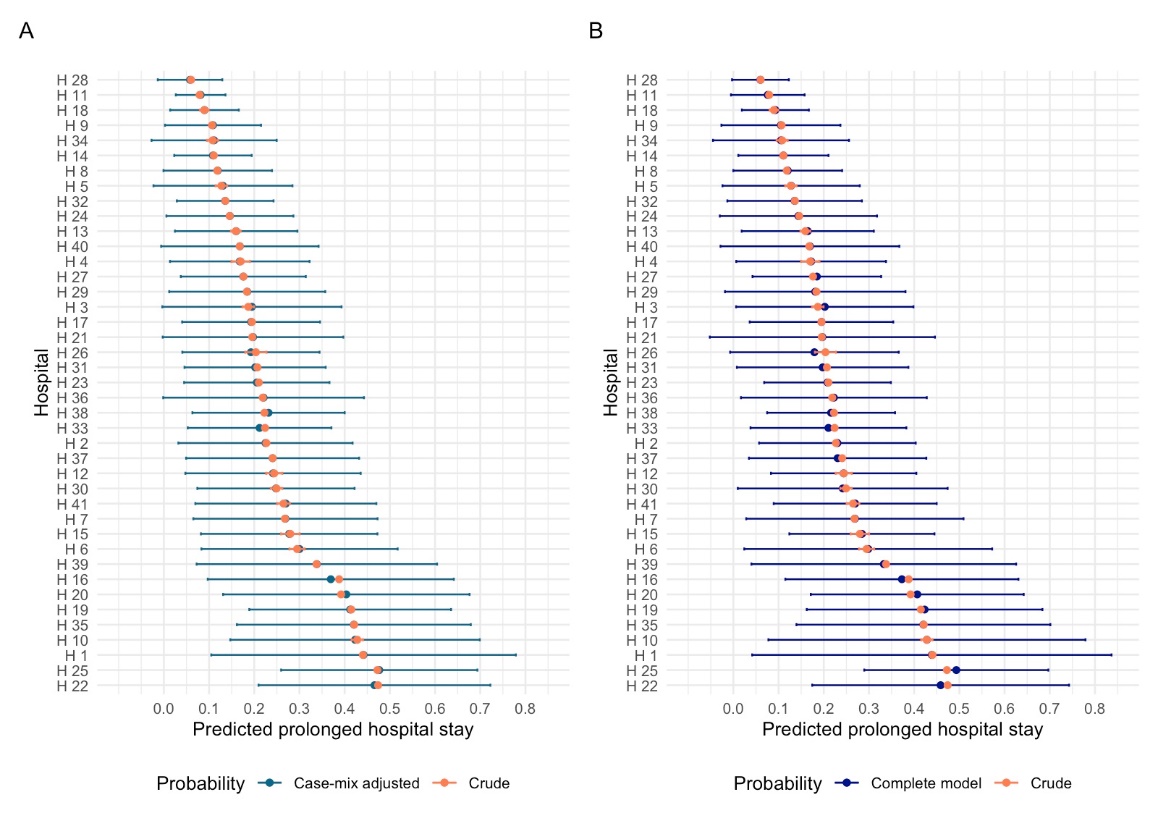
**

**Supplementary figure 7A-B** Estimated probabilities of readmissions per hospital A. Crude vs. adjustment for case-mix B Crude vs. adjustment for case-mix and treatment-related factors (*n* = 4990).

**
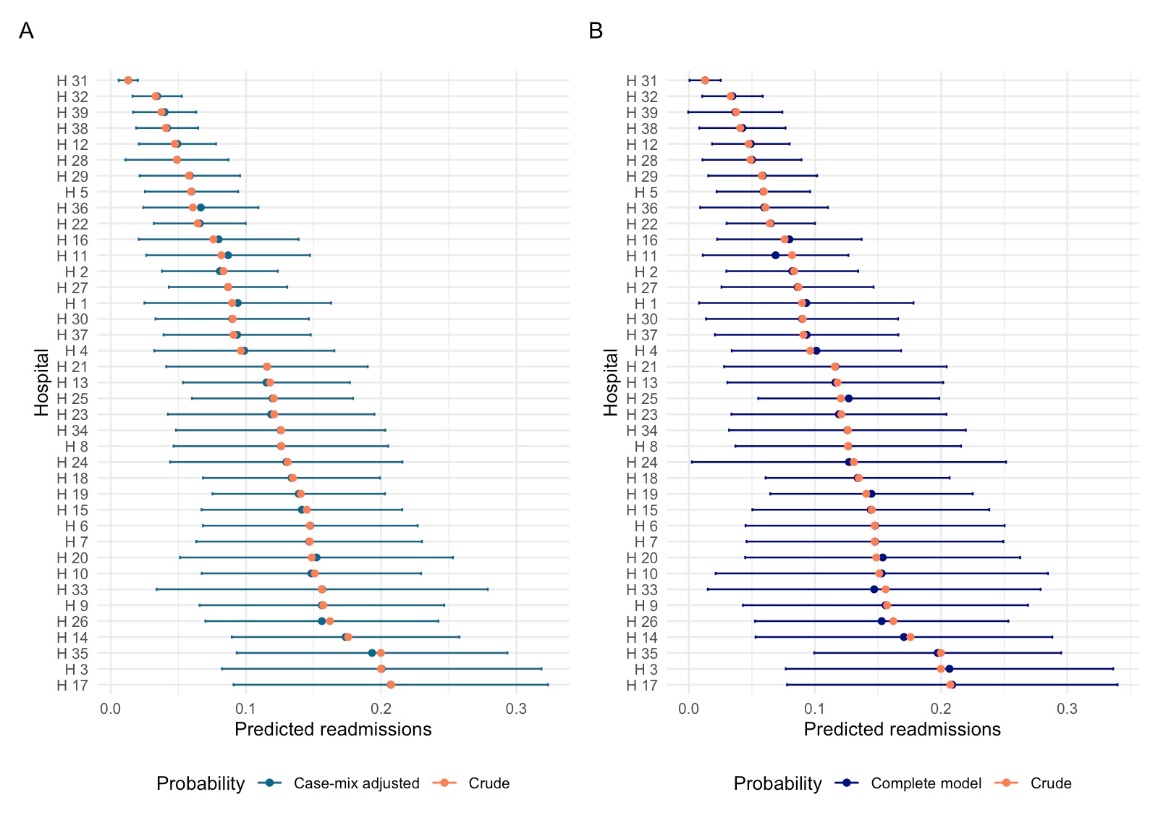
**

| **Supplementary table 1.** Marginal pseudo-R^2^ for all outcomes: the shown metrics are the increment of the pseudo-R^2^ when adding case-mix and treatment-related factors to the model. A score of 1 is equal to 100% variance explained. | | |
| --- | --- | --- |
| ***Outcome*** | Case-mix factors | Treatment factors |
|  | **Marginal pseudo-R**^2^ | **Marginal pseudo-R**^2^ |
| **>15 LN retrieved** | 0.059 | 0.081 |
| **Negative margin (R0)** | 0.317 | 0.026 |
| **Severe complications (≥ CD 3a)** | 0.065 | 0.027 |
| **Escalation of care^a^** | 0.103 | 0.018 |
| **Reoperation^b^** | 0.051 | 0.015 |
| **Proportion >14 DoH** | 0.079 | 0.043 |
| **Readmission (30-day)** | 0.031 | 0.209 |
| **30-day mortality** | 0.339 | 0.408 |
| *LN* Lymph nodes *DoH* Days of hospitalization *CD* Clavien-Dindo | | |
| Values are percentages, unless otherwise indicated. | | |
| ^a^ Unplanned readmission to a higher surveillance unit (either intermediate- or intensive care unit). | | |
| ^b^ Surgical intervention under general anesthesia. | | |

**Supplementary Figure 8.** Distribution of treatment probability in each given center based on all available case-mix characteristics.


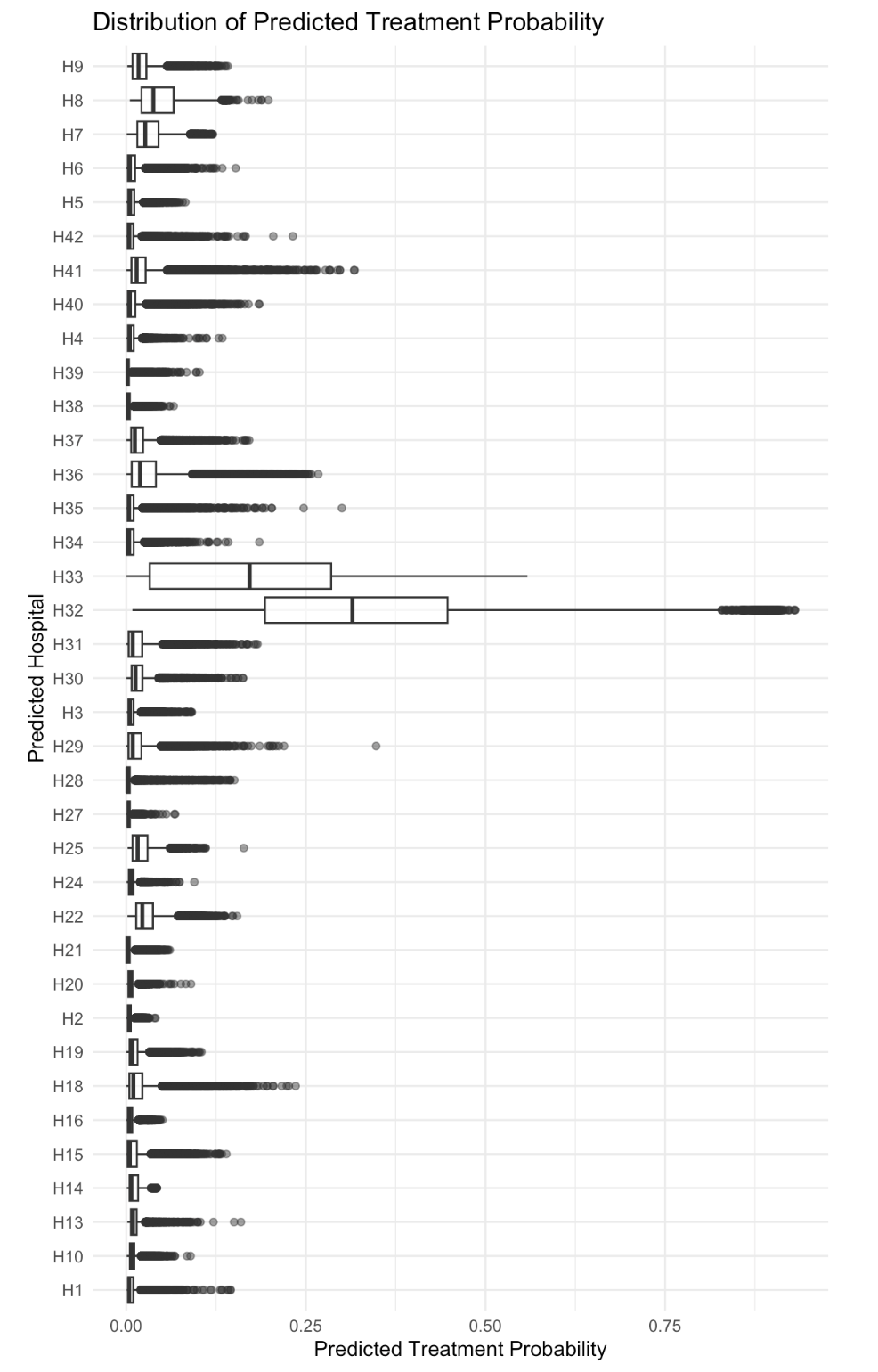


| **Supplementary table 2.** Marginal pseudo-R^2^ for all outcomes in the sensitivity analysis: the shown metrics are the increment of the pseudo-R^2^ when adding case-mix and treatment-related factors to the model. A score of 1 is equal to 100% variance explained. | | |
| --- | --- | --- |
| ***Outcome*** | Case-mix factors | Treatment factors |
|  | **Marginal pseudo-R**^2^ | **Marginal pseudo-R**^2^ |
| **>15 LN retrieved** | 0.069 | 0.076 |
| **Negative margin (R0)** | 0.335 | 0.022 |
| **Severe complications (≥ CD 3a)** | 0.062 | 0.015 |
| **Escalation of care^a^** | 0.095 | 0.028 |
| **Reoperation^b^** | 0.051 | 0.021 |
| **Proportion >14 DoH** | 0.075 | 0.023 |
| **Readmission (30-day)** | 0.030 | 0.272 |
| **30-day mortality** | 0.474 | 0.183 |
| *LN* Lymph nodes *DoH* Days of hospitalization *CD* Clavien-Dindo | | |
| Values are percentages, unless otherwise indicated. | | |
| ^a^ Unplanned readmission to a higher surveillance unit (either intermediate- or intensive care unit). | | |
| ^b^ Surgical intervention under general anesthesia. | | |
